# Positive and negative regulation of seed germination by the Arabidopsis GA hormone receptors, *GID1a*,* b*, and *c*

**DOI:** 10.1002/pld3.83

**Published:** 2018-09-21

**Authors:** Wenjing Ge, Camille M. Steber

**Affiliations:** ^1^ Department of Crop and Soil Science Washington State University Pullman Washington; ^2^ State Key Laboratory of Grassland Agro‐ecosystems School of Life Sciences Lanzhou University Lanzhou Gansu China; ^3^ Wheat Health, Genetics and Quality Unit USDA‐ARS Pullman Washington

**Keywords:** epistasis, germination, gibberellin, GID1, seed dormancy

## Abstract

Epistasis analysis of *gid1* single and double mutants revealed that *GID1c* is a key positive regulator of seed germination, whereas the *GID1b* receptor can negatively regulate germination in dormant seeds and in the dark. The *GID1* GA receptors were expected to positively regulate germination because the plant hormone gibberellin (GA) is required for seed germination in *Arabidopsis thaliana*. The three GA hormone receptors, GID1a, GID1b, and GID1c, positively regulate GA responses via GA/GID1‐stimulated destruction of DELLA (Asp‐Glu‐Leu‐Leu‐Ala) repressors of GA responses. The fact that the *gid1abc* triple mutant but not *gid1* double mutants fail to germinate indicates that all three GA receptors can positively regulate non‐dormant seed germination in the light. It was known that the *gid1abc* triple mutant fails to lose dormancy through the dormancy breaking treatments of cold stratification (moist chilling of seeds) and dry after‐ripening (a period of dry storage). Previous work suggested that there may be some specialization of *GID1* gene function during germination because *GID1b* mRNA expression was more highly induced by after‐ripening, whereas *GID1a* and *GID1c* mRNA levels were more highly induced by cold stratification. In light‐germinated dormant seeds, the *gid1b* mutation can partly rescue the germination efficiency of *gid1a* but not of *gid1c* seeds. Thus, *GID1b* can function as an upstream negative regulator *GID1c*, a positive regulator of dormant seed germination. Further experiments showed that *GID1b* can negatively regulate dark germination. Wild‐type Arabidopsis seeds do not germinate well in the dark. The *gid1b* and *gid1ab* double mutants germinated much more efficiently than wild type, *gid1c*, or *gid1ac* mutants in the dark. The observation that the *gid1ab* double mutant also shows increased dark germination suggests that *GID1b*, and to some extent *GID1a*, can act as upstream negative regulators of *GID1c*. Since the *gid1abc* triple mutant failed to germinate in the dark, it appears that *GID1c* is a key downstream positive regulator of dark germination. This genetic analysis indicates that the three *GID1* receptors have partially specialized functions in GA signaling.

## 1 INTRODUCTION

Plant embryos can survive for extended periods of time in a dry seed, in a state resembling suspended animation (reviewed in Bewley, Bradford, Hilhorst, & Nonogaki, 2013). This allows plants to be spread by wind or animal activity, and allows humans to store crop varieties for extended periods as metabolically quiescent dry seeds. The seeds of many temperate plant species are dormant at maturity, meaning that they cannot germinate unless they experience dormancy‐breaking conditions. In Arabidopsis, seed dormancy can be released by a period of dry storage called after‐ripening or by cold stratification, taking up or imbibing water in the cold. Seed germination is a process beginning with water uptake and ending with the final germination event when the embryonic root penetrates the surrounding structures of the seed coat. Seed dormancy allows time for seed dispersal, prevents seeds from germinating out of season since a spring annual must experience an extended period of wintry cold stratification to germinate, and can help species to survive natural disasters as seeds stored in the soil.

It is well established that seed dormancy and germination are regulated by the opposing action of two plant hormones, abscisic acid (ABA) and gibberellin (GA) (reviewed by Finkelstein, Reeves, Ariizumi, & Steber, 2008). ABA is needed to establish dormancy during seed maturation, maintains dormancy in mature seeds, and can inhibit germination when externally applied to seeds. GA stimulates seed germination and is absolutely required for germination in some plant species like Arabidopsis and tomato. GA also stimulates stem elongation, the transition to flowering, and fertility. Arabidopsis seeds require light to germinate efficiently. Red light breaks Arabidopsis seed dormancy while far‐red light promotes dormancy. GA and ABA also participate in light regulation of germination. Red light stimulates GA biosynthesis and inhibits ABA biosynthesis, whereas far‐red light promotes GA turnover and stimulates ABA biosynthesis (reviewed by Yamaguchi, 2008). In Arabidopsis, the *ga1* biosynthesis mutant completely fails to germinate unless GA hormone is exogenously applied or the seed coat is cut. Moreover, GA‐insensitive signaling mutants like *sly1* (*sleepy1*) cause increased seed dormancy, whereas GA hypersensitive mutants like *spy* (*spindly*) cause decreased seed dormancy.

GA stimulates seed germination and other developmental events by lifting DELLA protein repression of GA responses (reviewed by Hauvermale, Ariizumi, & Steber, 2012; Thomas, Blázquez, & Alabadi, 2016; Urbanova & Leubner‐Metzger, 2016). DELLA proteins are named for a conserved amino acid sequence (Asp‐Glu‐Leu‐Leu‐Ala), and are a family of nuclear‐localized transcriptional regulators that negatively regulate GA signaling and responses. The partially specialized roles of the five DELLA family members in Arabidopsis have been defined by characterizing how loss of function mutations in DELLA genes suppress *ga1‐3* phenotypes (Cheng et al., 2004; Dill, Jung, & Sun, 2001; King, Moritz, & Harberd, 2001; Lee et al., 2002; Tyler et al., 2004; Yu et al., 2004; Wild et al., 2012). For example, loss of the DELLA gene *RGL2* rescued the germination of the GA biosynthesis mutant *ga1‐3* in the light, but complete rescue of *ga1‐3* dark germination required mutations in DELLA genes *RGL2*,* RGA*, and *GAI*. Thus, *RGL2* is considered to be the main DELLA negatively regulating germination. *RGA* and *GAI* are the main DELLAs negatively regulating cell expansion, whereas *RGA*,* RGL1*, and *RGL2* negatively regulate the transition to flowering. DELLA *RGL3* interacts with jasmonic acid signaling to regulate plant defense responses.

GA lifts DELLA repression of seed germination and other GA responses by directing DELLA destruction via the ubiquitin‐proteasome pathway (Ariizumi & Steber, 2007; McGinnis et al., 2003; Nelson & Steber, 2016; Tyler et al., 2004). The GA hormone signal is perceived by the GIBBERELLIN‐INSENSITIVE DWARF1 (*GID1*) GA receptors (Nakajima et al., 2006; Ueguchi‐Tanaka et al., 2005). In the absence of GA, DELLA proteins repress seed germination and other GA responses. GA binding to the GID1 receptor results in a conformational change in the receptor, enabling it to bind DELLA protein. Formation of the *GID1*‐GA‐DELLA complex, causes the SLY1 F‐box protein to bind the DELLA protein (Ariizumi, Lawrence, & Steber, 2011; Wang & Deng, 2011). SLY1 is the F‐box subunit of an SCF E3 ubiquitin ligase that catalyzes DELLA polyubiquitination, causing DELLA to be degraded by the 26S proteasome. DELLA destruction allows seeds to germinate.

Since *GID1* and *SLY1* are both positive regulators of GA signaling, loss of function should result in increased seed dormancy/reduced germination. The *sly1* mutants are GA‐insensitive dwarves that exhibit increased seed dormancy (Ariizumi & Steber, 2007). The *sly1‐2* allele requires one to two years to after‐ripen, whereas the stronger *sly1‐t2* fails to after‐ripen. Rescue of *sly1‐2* germination by after‐ripening and by overexpression of the *GID1* receptor genes on the CaMV 35S promoter is associated not with decreased DELLA protein accumulation, but with increased formation of the GID1‐DELLA protein complex (Ariizumi et al., 2013; Fukazawa, Ito, Kamiya, Yamaguchi, & Takahashi, 2015). It appears that GID1‐binding to DELLA blocks DELLA interaction with downstream regulators of GA signaling.

The *GID1a*,* GID1b*, and *GID1c* genes appear to function redundantly as positive regulators of seed germination in the light (Griffiths et al., 2006; Iuchi et al., 2007; Voegele, Linkies, Mueller, & Leubner‐Metzger, 2011; Willige et al., 2007). The fact that the *gid1a gid1b gid1c* triple mutant is unable to germinate unless the seed coat is cut, while the *gid1* single mutants can germinate suggests that the three genes act redundantly to stimulate germination. Moreover, a positive role in seed germination is suggested by the fact that overexpression of each Arabidopsis *GID1* gene increases *sly1* mutant germination and increases the GA‐sensitivity of *ga1‐3* during germination (Ariizumi et al., 2013; Hauvermale, Ariizumi, & Steber, 2014; Hauvermale, Tuttle, Takebayashi, Seo, & Steber, 2015). Interestingly, overexpression of the *GID1b* gene more strongly stimulated germination than overexpression of the *GID1a* or *GID1c* genes. This suggested that there might be some specialization in *GID1* gene function in seed germination.

In order to examine whether there may be more specialization in *GID1* gene function during seed germination, this study closely examined the relative effects of *gid1* single and double mutants on initial *Arabidopsis* seed dormancy, dormancy loss, and germination. We reasoned that conditions likely to inhibit germination would accentuate the germination phenotype of *gid1* single and double mutants allowing us to better examine their relative effects on seed dormancy and germination. Voegele et al. (2011) provided a careful examination of the germination phenotypes of after‐ripened *gid1* mutants germinated in the light. Using the same *gid1* alleles, this study examined the effect of *gid1* single and double mutants on initial dormancy, after‐ripening, dark germination, and GA sensitivity. Examination of mutant phenotypes told a new story about GID1 regulation of germination. *GID1b* appeared to behave like a negative regulator of germination in dormant seeds germinated in the light and in dark‐germinated seeds. *GID1c* is a strong positive regulator of germination, whereas the role of *GID1a* appears to depend upon dormancy and lighting conditions.

## 2 MATERIALS AND METHODS

### 2.1 Plant materials and growth conditions

The *gid1a‐1*,* gid1b‐1*, and *gid1c‐2* single mutants, double mutants, and segregating triple mutant (*gid1b/gid1b gid1c/gid1c gid1a/+*) in the *Arabidopsis thaliana* ecotype Columbia (Col‐0) background were a gift from Claus Schwechheimer (Willige et al., 2007). Each of these three alleles contains a T‐DNA insertion in the major second exon of the *GID1* gene, predicted to result in a loss‐of‐function. All genotypes were confirmed by PCR using Taq (NEB) and the primers reported by Willige et al., 2007. Genomic DNA was prepared according to McKinney et al., 1995, and PCR was performed using the PCR program: 3 min at 95°C, 42 cycles of 1 min at 95°C, 50 s at the annealing temperature, 1 min at 72°C, then 10 min at 72°C. To make sure that seeds used were exposed to the same environment during development and were of similar age, all *gid1* mutants and the Columbia (Col‐0) wild type (WT) were grown side‐by‐side in a Conviron growth chamber under a 16 hr day under fluorescent lamps at 200 μmol m^−2^ sec^−1^ at 22°C. The accession numbers for *GID1a*,* GID1b*, and *GID1c* are AT3G05120, AT3G63010, and AT5G27320, respectively.

In order to obtain high initial seed dormancy, seeds were harvested from plants near physiological maturity, when 40%–60% of the siliques were yellow but some were still green (as in Nelson, Ariizumi, & Steber, 2017). When such plants were hand threshed, only brown mature seeds, not green seeds, were obtained. Experiments were performed using two independent sets of seeds, and the second set of seeds had more initial dormancy than the first. For dry after‐ripening (AR), seeds were stored in open tubes at room temperature (~22°C) and 15%–20% humidity for 2 days (d), 1 week (wk), 2 weeks, or 4 weeks. Light germination experiments were plated immediately upon reaching the desired after‐ripening time point. The dark germination plating experiments were performed using seed stored at −20°C for less than 2 months to maintain dormancy.

### 2.2 Light germination

For all germination experiments, 80–120 seeds were sterilized with 10% (v/v) bleach and 0.2% (v/v) Triton X‐100 for 10 min, and washed six times with sterile deionized water. Seeds were plated on MS‐agar plates containing 0.8% (w/v) agar and 0.5× Murishige and Skoog salts (MS, Sigma‐Aldrich), and germination was scored daily for 7 days (d) during incubation under fluorescent light (60 μmol m^−2^ s^−1^) at 22°C. For the GA dose–response experiments, 2 and 4 weeks (wk) after‐ripened seeds were incubated with gentle agitation in 10 μM paclobutrazol (PAC) in 0.5× MS salts and 5 mM MES (2‐(N‐morpholino) ethanesulfonic acid), pH 5.5; Sigma) for 48 hr in dark at 4°C to suppress endogenous GA biosynthesis, then washed 6 times with sterile water before plating on MS‐agar containing 0 μM, 0.01 μM, 0.1 μM and 0.5 μM gibberellin A_3_ (GA_3_, Phytotechnology) Germination was scored daily for 5 days and averaged over 4 biological replicates.

### 2.3 Dark germination

For dark germination experiments, 2 weeks and 4 weeks after‐ripened (AR) seeds were sterilized under 0.73 μmol m^−2^ s^−1^ light followed by plating under multiple fluorescent light intensities of: (a) Bright light of 16.9 μmol m^−2^ s^−1^, (b) Dim light of 1.3 μmol m^−2^s^−1^, and (c) Dimmer light of 0.3 μmol m^−2 ^s^−1^. Cool white 40W fluorescent lamps were used to generate varying light intensities (Sylvania FO30/841/XP/SS/ECO3 and FO40/741/ECO). Seeds were also plated under a green LED lamp (Plant Safe 9‐LED Mini Flashlight, Hydroponics Nation) with a light intensity of 1.0 μmol m^−2^ s^−1^. Plates were wrapped with two layers of aluminum foil (one silver sheet and one black sheet), then incubated at 22°C for 14 days in the dark. Germination was scored only once under a dissection microscope. The average germination rate was calculated using at least three independent replicates. An ANOVA was performed in SPSS v19.0 to determine which germination differences were significant as shown using letters for statistical classes in Supporting Information Figure S2.

### 2.4 Determining seed size

Seed size was determined using two independent sets of *gid1* single and double mutant, and of Col WT growth side‐by‐side in two greenhouse experiments. For each set, seeds of each genotype were pooled. We stochastically selected three subsamples of 500 seeds from each set of the pooled samples. Seeds were counted under a dissection microscope (Leica), and then the mass of each subsample determined using a quantitative balance (Denver Instruments).

## 3 RESULTS

### 3.1 Effects of *gid1* single and double mutants on light germination

**Figure 1:**
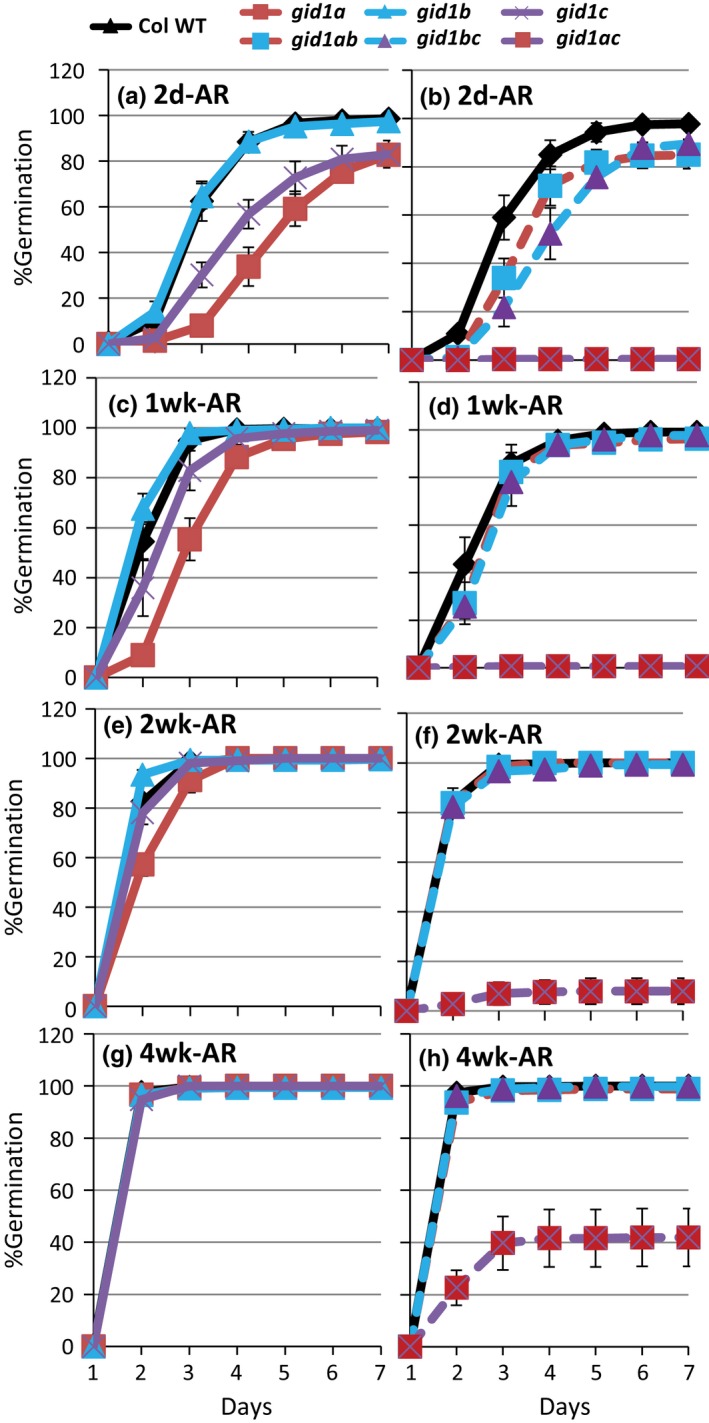
After‐ripening time course of *gid1* germination. An after‐ripening time course compared *gid1* single (a,c,e,g) and double (b,d,f,h) mutant seed germination. Percentage germination was scored daily over 7 days imbibition on 0.5× MS‐agar at 22°C, over four dry after‐ripening time points (1 days, 1 week, 2 weeks and 4 weeks). Mean germination is shown with error = *SD*,* n* = 3

The effect of *gid1* single and double mutants on dormancy and after‐ripening were examined by comparing light germination to Col WT at 2 days, 1, 2, and 4 weeks of dry after‐ripening. The *gid1a* and *gid1c*, but not *gid1b*, single mutants showed higher initial dormancy than WT (Figure 1a). Of the double mutants, *gid1ac* had the strongest seed dormancy, showing a complete failure to germinate through 2 weeks after‐ripening (Figure 1b). All mutants showed increasing germination with after‐ripening (Figure 1a–h). The *gid1ac* double mutant reached 41% germination by 4 weeks after‐ripening. To determine if *gid1ac* seeds were viable, seeds coats were cut at 2 weeks‐AR resulting in 86% germination within 5 days of imbibition. Since embryos are sometimes injured when the seed coat is cut, this suggests that the *gid1ac* seeds are dormant but viable. This is consistent with previous work showing that the *gid1ac* double mutant seeds are viable based on tetrazolium staining (Voegele et al., 2011). These results suggest that *GID1a* and *GID1c* are the main GA receptors serving as positive regulators of germination in the light. Previous work showed that the *gid1a gid1b gid1c* triple mutant fails to germinate and does not after‐ripen (Willige et al., 2007). However, no single or double *gid1* mutant is sufficient to prevent after‐ripening, suggesting that the three *GID1* genes act additively to positively regulate seed germination and dormancy loss.

**Figure 2:**
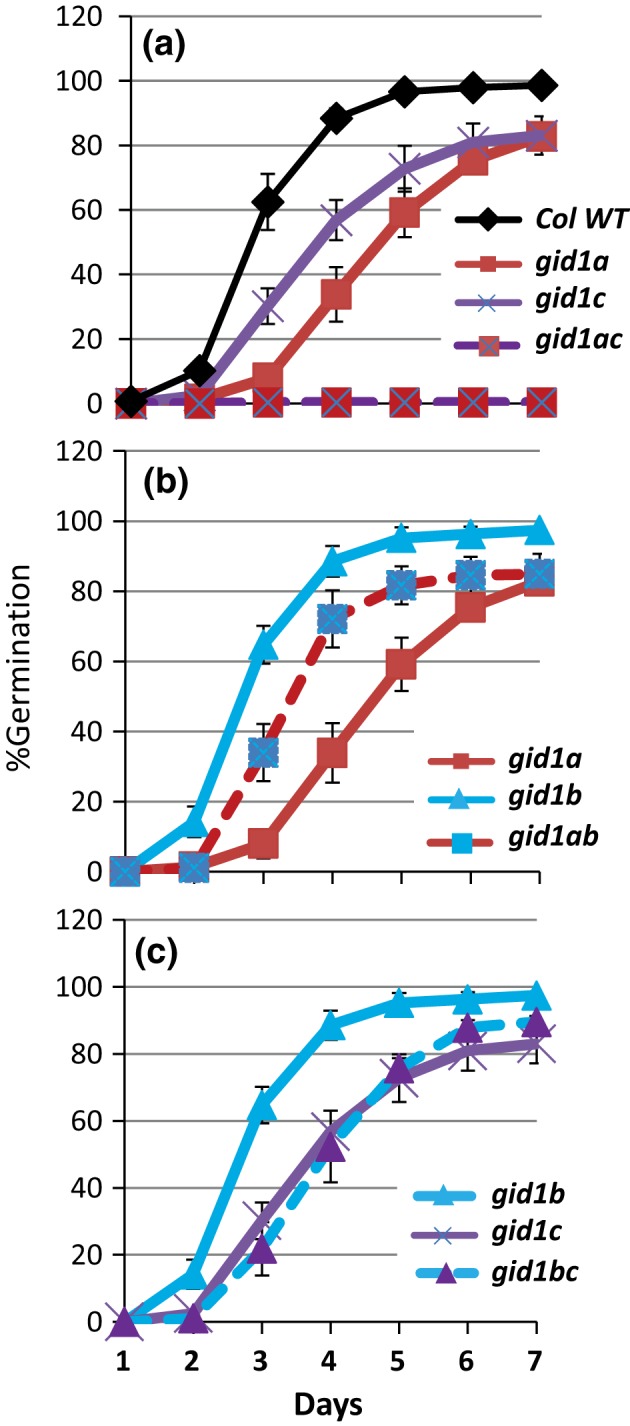
Epistasis analysis of dormant *gid1* seed germination in the light. Compared are: (a) *gid1a* and *gid1c* to *gid1ac*, (b) *gid1a* and *gid1b* to *gid1ab*, and (c) *gid1a* and *gid1c* to *gid1ac*. Seeds were germinated at 2 days AR (as in Figure 1a,b). Error = *SD*,* n* = 3

Another story about the relative roles of *GID1a*,* GID1b*, and *GID1c* emerged from an epistasis analysis comparing single mutant parents to corresponding double mutant germination at 2 days after‐ripening. Consistent with the notion that *GID1a* and *GID1c* both positively regulate seed germination, the *gid1a* and *gid1c* mutations had an additive effect on seed germination. The *gid1a gid1c* double mutant failed to germinate, whereas the *gid1a* mutant showed 33.9% and that *gid1c* mutant 56.8% germination with 4 days incubation (Figure 2a). If *GID1b* were a weak positive regulator of seed germination, then you would expect the *gid1bc* and *gid1ab* germination to be similar to or reduced compared to the *gid1c* and *gid1a* parents. The *gid1bc* double mutant germination (52.3%) was similar to *gid1c* (56.8%), but lower than *gid1b* germination (88.5%) at 4 days incubation (Figure 2c). Thus, *gid1c* is epistatic to *gid1b*. In contrast, the *gid1ab* double mutant had a germination phenotype that was intermediate (72.1%) between *gid1a* (33.9%) and *gid1b* (88.5%) at 4 days incubation (Figure 2b). Thus, the *gid1b* mutation appears to partly rescue *gid1a* germination, suggesting that *GID1b* acts as a negative regulator of germination in dormant seeds. The observation that *gid1b* and *gid1a* have additive effects suggests that they act in parallel. The fact that *gid1b* can partly rescue *gid1a* but not *gid1c* germination suggests that *GID1b* functions upstream of *GID1c* as a negative regulator of seed germination. The *gid1ab* germination appears depend on stimulation of germination by *GID1c*.

### 3.2 The effect of *gid1* single and double mutants on GA dose–response during light germination

*GID1* GA receptor mutants were expected to have decreased ability to respond to GA during seed germination. The relative effects of the *gid1* single and double mutants on GA dose–response curves were examined. The GA biosynthesis inhibitor paclobutrazol (PAC) was used to suppress endogenous GA biosynthesis in WT and *gid1* mutant seeds before plating on increasing concentrations of GA_3_ (Supporting Information Figure S1 ABCD). The *gid1b* mutant showed a very similar GA dose–response to Col WT. The *gid1a* and *gid1c* single mutants and the *gid1ac* double mutant showed the strongest decrease in GA dose–response. The relative GA dose–response curves of the *gid1* single and double mutants were similar at 2 and 4 weeks of after‐ripening.

**Figure 3:**
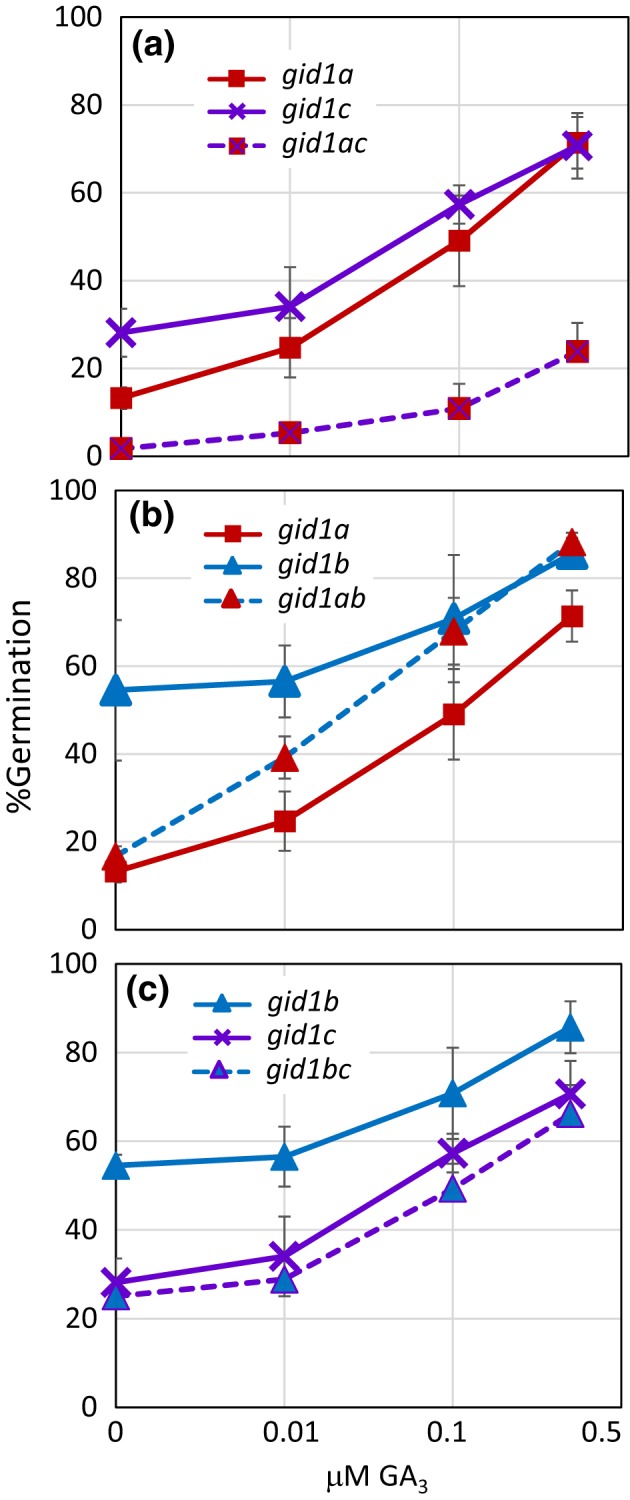
Epistasis analysis of *gid1* mutant GA dose–response. Compared are light germination of: (a) *gid1a* and *gid1c* to *gid1ac*, (b) *gid1a* and *gid1b* to *gid1ab*, and (c) *gid1a* and *gid1c* to *gid1ac*. Seeds were incubated in 10 μM PAC for 48 hr at 4°C in dark, washed, and then incubated in the light for 5 days at 22°C on MS‐agar containing 0, 0.01, 0.1 and 0.5 μM GA_3_. Germination was scored daily. The *x*‐axis is log_10_ scale. Error = *SD* (*n* = 4) is shown

An epistasis analysis was conducted comparing *gid1* double mutant GA sensitivity to single mutant parents at 4 weeks after‐ripening (Figure 3). The *gid1a* and *gid1c* mutations had an additive effect, such that the *gid1ac* double mutant was less GA sensitive than the parents (Figure 3a). The *gid1bc* double mutant did not differ significantly from the *gid1c* mutant which was less GA sensitive than the *gid1*b mutant (Figure 3c). The *gid1ab* double mutant phenotype varied with GA concentration, appearing similar to *gid1a* at 0 μM GA, intermediate between *gid1a* and *gid1b* at 0.01 μM, and similar to *gid1b* at 0.1 and 0.5 μM GA (Figure 3b).

### 3.3 The effect of *gid1* single and double mutants on dark germination

We expected germination in the dark to exacerbate the *gid1* seed dormancy phenotypes, making it easier to compare the relative effects of *gid1* single and double mutants on germination capacity. Experiments were conducted at 2 and 4 weeks of after‐ripening using seeds that had been plated under varying light conditions referred to as: bright (16.9 μmol m^2 ^s^−1^), dim (1.3 μmol m^−2 ^s^−1^), and dimmer (0.3 μmol m^−2 ^s^−1^) fluorescent light, and green light (1.0 μmol m^−2^s^−1^). Seed germination was scored after dark incubation for 14 days at 22°C. While similar trends were observed at 2 and 4 weeks of after‐ripening, the 4 weeks after‐ripening data are described in more detail because it is easier to observe significant differences when the germination rates are higher (Supporting Information Figure S2AB; Supporting Information Table S1; Supporting Information Table S2). The effects of *gid1* mutants on dark germination varied with light intensity during plating. Generally, higher fluorescent light intensity during plating stimulated dark germination in WT and in all *gid1* mutants, and green light resulted in the least efficient seed germination (Supporting Information Figure S3).

**Figure 4:**
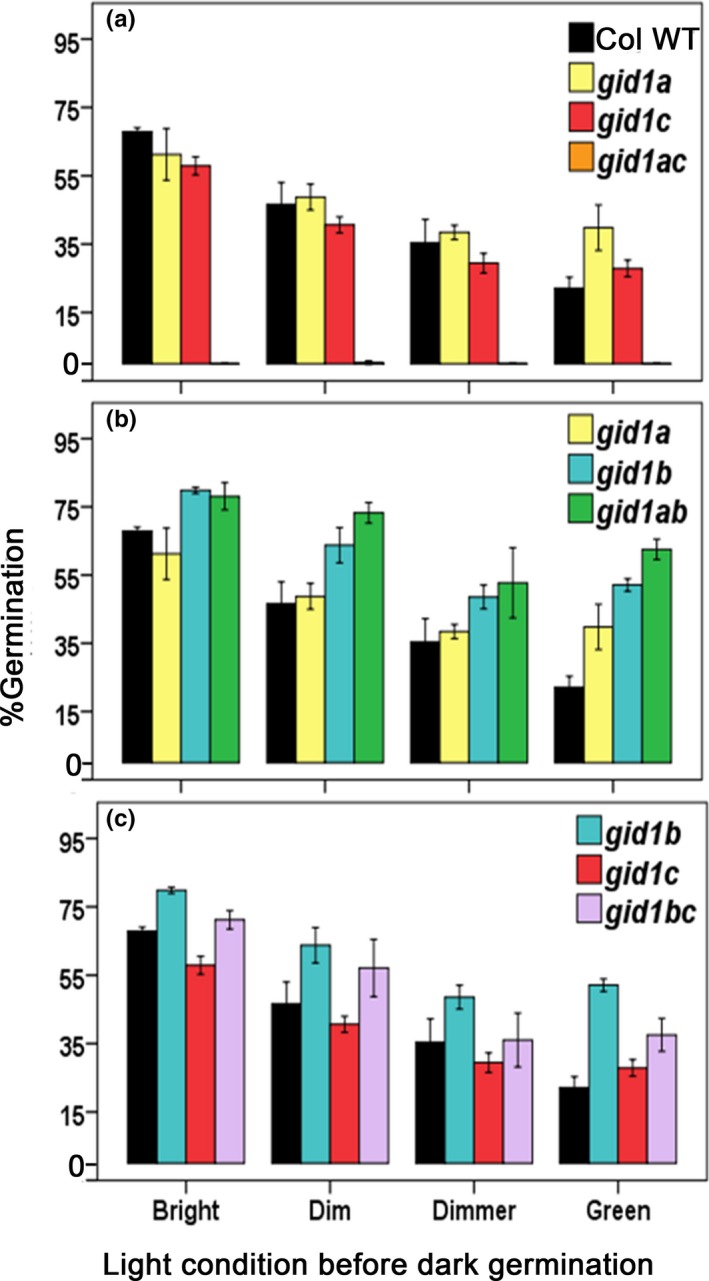
Epistasis analysis of the *gid1* dark germination phenotype. Compared in order are dark germination of: (a) *gid1a* and *gid1c* to *gid1ac*, (b) *gid1a* and *gid1b* to *gid1ab*, and (c) *gid1a* and *gid1c* to *gid1ac*. Seeds were plated at 4 weeks AR. The *x*‐axis labels “Bright”, “Dim”, “Dimmer” indicate the fluorescent light intensity or quality during plating prior to incubation for 14 days in the dark at 22°C. Shown is the mean ±*SE* (*n* = 3)

Many of the effects of the *gid1* single and double mutants were observed regardless of lighting during plating, although some effects were clearer under one or more condition. Of the single mutations, only *gid1c* showed a significant decrease in dark germination compared to WT at 4 weeks AR and only after plating under bright lighting (Figure 4; Supporting Information Figure S2). Surprisingly, *gid1b* consistently germinated more efficiently than Col WT in the dark. The *gid1a* mutation did not show a significant decrease in germination compared to Col WT, and actually germinated more efficiently than WT when 4 weeks AR seeds were plated under green light. Thus, *GID1c* behaves like a strong positive regulator and *GID1b* a negative regulator, while *GID1a* behaves as a weak positive or negative regulator of dark germination depending on conditions.

An epistasis analysis of dark germination was made by comparing double mutants to single mutant parents (Figure 4). The experiment was repeated using a second batch of seeds that had higher initial dormancy (Supporting Information Figure S4). The *gid1* mutants showed similar effects and epistasis relationships in the two experiments. The *gid1ac* double mutant failed to germinate in the dark and, therefore, germinated less efficiently than either *gid1a* or *gid1c* mutants (Figure 4; Supporting Information Figure S4). This additive effect suggests that *GID1c* and *GID1a* act in parallel as positive regulators of dark germination. Interestingly, the *gid1ab* double mutant germinated more efficiently than either the *gid1a* or *gid1b* single mutants when plated under green or dim lighting conditions, suggesting that *GID1a* can also act as a negative regulator of dark germination. Because the second set of seeds had more initial dormancy, it was more apparent that the *gid1a* mutation mildly stimulated germination, and that the *gid1ab* double mutant germinated more efficiently than the *gid1b* single mutant. Thus, *GID1a* is a weak negative regulator of dark germination in the presence of *GID1c*. Thus, *GID1c* is a strong positive regulator of seed germination that appears to be negatively regulated by *GID1b* and *GID1a*. The role of *GID1a* appears to be context dependent, acting as a positive regulator when *GID1b* is present and acting as a negative regulator when *GID1c* is present. These results strongly suggest that the Arabidopsis GA receptors have specialized roles in dark seed germination. The *gid1bc* double mutant germinated more efficiently than *gid1c* but less efficiently than *gid1b*. This suggests that *GID1c* and *GID1b* act in parallel to positively and negatively regulate dark germination, respectively. If *GID1b* and *GID1c* do act in parallel, then we might expect the *gid1abc* triple to germinate in the dark in a manner similar to *gid1ab*.

**Figure 5:**
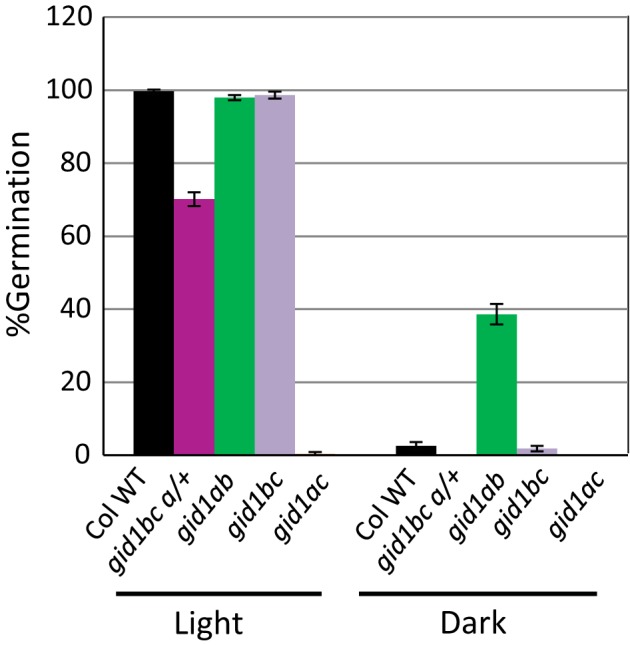
Light and dark germination of the *gid1abc* segregating line. Seeds of Col WT,* gid1ab*,* gid1ac*,* gid1bc*, and of the *gid1 b/b c/c a/+* line segregating for *gid1a*, were plated at 4 weeks‐AR under fluorescent lights (17 μmol m^−2^ s^−1^), then germinated either in the dark or under lights at 22°C. Error bars indicate *SD* (*n* = 3)

Previous work showed that the *gid1abc* triple mutant fails to germinate in the light unless germination is rescued by cutting the seed coat (Willige et al., 2007). However, the effect of the *gid1abc* triple mutation on dark germination is unknown. Since the *gid1abc* triple homozygous mutant is infertile, seeds of the *gid1 b/b c/c a/+* line that segregates for the *gid1abc* triple homozygous mutant were plated to compare dark and light germination phenotypes (Figure 5). Seeds of wild‐type Col, *gid1bc*,* gid1ab*, and *gid1ac* were included for comparison. Seeds were sterilized and plated under fluorescent (17 μmol m^−2^ s^−1^) lights and then incubated at 22°C either under lights or in the dark. The *gid1b/b c/c a/+* line should segregate 25% *gid1abc* triple homozygous mutant, 50% *gid1b/b c/c a/+* heterozygotes, and 25% *gid1b/b c/c* double homozygous mutant. This line was expected to show about 75% germination under lights, and actually showed 70% germination. In the dark, the segregating line showed 0% germination, Col WT and *gid1bc* about 2% germination, *gid1ac* 0% germination, and *gid1ab* 39% germination. If the *gid1abc* triple germinated as well as the *gid1ab* double, then the segregating line should show about 10% germination (25% × 39%). Thus, the dark germination of the *gid1abc* triple does not resemble the *gid1ab* double mutant, indicating that *GID1c* must be present for *gid1b* and *gid1a* mutations to rescue dark seed germination. Thus, *GID1c* appears to act downstream of *GID1b* and *GID1a* to stimulate seed germination, and *GID1b* and *GID1a* appear to negatively regulate germination via *GID1c*.

### 3.4 The effect of *gid1* single and double mutants on seed size

**Figure 6:**
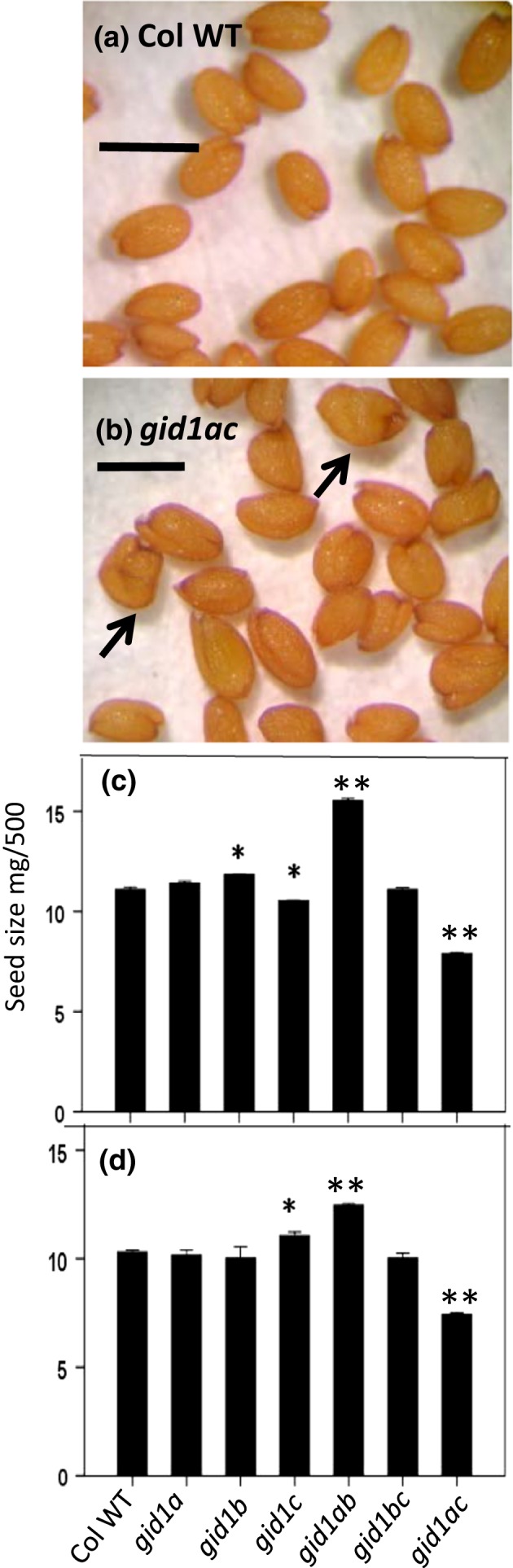
Effect of *gid1* genotype on seed size. Comparison of: (a) Col WT and (b) *gid1ac* seed morphology showing that *gid1ac* seeds appear shriveled. Seed size was evaluated in two independent sets of seeds, (c) Set 1 and (d) Set 2. Student's *t* test was used to examine the statistical significance of seed size differences from WT, *p < 0.05, **p < 0.001. The bar indicates 0.5 mm

While performing germination experiments, we observed some morphological differences in the *gid1* mutant seeds themselves. For example, the *gid1ab* seeds appeared to be larger than Col WT, whereas the *gid1ac* double mutant seeds appeared somewhat shriveled (Figure 6a,b). To examine whether there was a quantitative difference in seed size, we measured the dry weight of 500 seeds of Col WT, the *gid1* single, and *gid1* double mutants in the two sets of seeds harvested at different times (Figure 6c,d). The *gid1ab* double mutant seeds were significantly larger and the *gid1ac* double mutant, significantly smaller than WT seeds in both experiments (*p* < 0.001). Thus, *GID1* genes appear to regulate Arabidopsis seed size, suggesting that they play a role during seed development as well as during seed germination.

## 4 DISCUSSION

**Figure 7:**
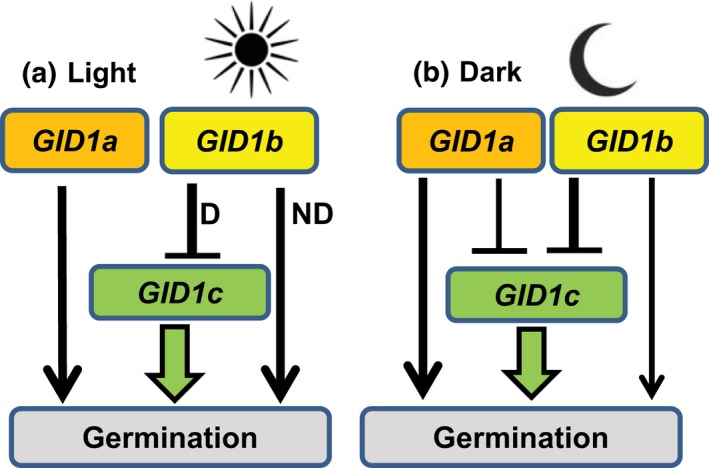
Model for *GID1* regulation of seed germination. (a) In the light, *GID1c* and *GID1a* act in parallel as positive regulators of seed germination. *GID1b* acts as a positive regulator of seed germination in nondormant (ND) seeds, but as a negative regulator of *GID1c* and seed germination in dormant (D) seeds. (b) In the dark, *GID1c* is the stronger and *GID1a* the weaker positive regulator of germination. Both *GID1b* and *GID1a* can negatively regulate germination via negative regulation of *GID1c*

GA hormone stimulates germination in many plant species, and it is known that GA hormone biosynthesis is required for germination in Arabidopsis and tomato (reviewed in Finkelstein et al., 2008). Based on this fact, positive regulators of GA signaling like the F‐box protein *SLY1* and the *GID1* GA receptors are expected to function as positive regulators of seed germination. The Arabidopsis *GID1a*,* GID1b*, and *GID1c* genes can all function as positive regulators of seed germination because the GA‐insensitive *gid1a gid1b gid1c* triple mutant cannot germinate and fails to after‐ripen (Willige et al., 2007). Epistasis analysis of germination under multiple conditions, however, revealed that some of the *GID1* receptors can behave as negative regulators of seed germination depending upon the genetic, developmental, and environmental context. *GID1c* functions as a positive regulator of seed germination in both the light and in the dark. *GID1a* functions as a positive regulator of seed germination in the light, but can behave like a mild negative regulator of *GID1c* and seed germination in the dark. *GID1b* appears to function as a negative regulator of seed germination in the dark and in dormant seeds germinating in the light when *GID1c* is present (Figure 7).

The *gid1ab* and *gid1ac* double mutants also reproducibly impacted seed size and morphology. This is interesting given that previous work suggested a role for *GID1* in control of ovule development (Ferreira, et al., 2008). The *gid1ab* double mutant produced larger seeds than WT (Figure 6). Larger seed size is sometimes associated with higher germination efficiency, but it is more often associated with better seedling emergence and vigor, presumably due to the presence of more stored reserves (Chaisurisri, Edwards, & El‐Kassaby, 1992; Edwards & Hartwig, 1971; Gómez, 2004; Leishman, Wright, Moles, & Westoby, 2000; Wu & Du, 2007). Increased seed size alone is not sufficient to explain the increased dark germination, since the *gid1a* and *gid1b* single mutants did not consistently increase seed size but did increase in dark germination compared to WT (Figure 4; Figure 6). It is possible, however, that the larger *gid1ab* double mutant seeds better supported seedling growth in the dark by providing more stored reserves to compensate for lack of photosynthesis. The *gid1ac* double mutant seed appears to be viable based on the fact that germination was rescued by nicking the seed coat and based on tetrazolium staining by Voegele et al., 2011;. However, *gid1ac* mutants produced smaller, shriveled seeds (Figure 6). The shriveled phenotype of the *gid1ac* double mutant, while not as extreme, was reminiscent of the shriveled seed phenotype observed in the ZHOUPI (ZOU) and INDUCER OF CBF EXPRESSION 1 (ICE1) bHLH transcription factor mutants (Denay et al., 2014). The *zou* and *ice1* mutants fail to degrade the endosperm during seed development, leading to a decrease in embryo expansion. The *gid1ac* embryos appeared to have developed to a normal shape and size when the seed coat was cut to stimulate germination. Future work will need to examine if *gid1ac* causes altered endosperm development.

Previous studies of loss‐of‐function alleles indicated that *GID1a*,* GID1b*, and *GID1c* act additively as positive regulators of seed germination in the light, but suggested that one *GID1* gene or another may play a more prominent role (Iuchi et al., 2007; Voegele et al., 2011; Willige et al., 2007). This study examined the same *gid1* mutant alleles used in both the Willige et al., 2007 and Voegele et al., 2011 studies. In the Voegele et al., 2011 study, the after‐ripened *gid1c* single mutant had a slightly lower, and *gid1b* a slightly higher, germination rate than Col WT. But those differences were not significant. Similarly, we found that none of the *gid1* single mutants had a statistically significant effect on germination when after‐ripened for 4 weeks (Figure 1). In seeds showing mild dormancy (2 day after‐ripened), however, the *gid1b* mutation was able to improve *gid1a* germination efficiency, but not *gid1c* germination in the light (Figure 2). Thus, loss of *GID1b* as a negative regulator of dormant seed germination can stimulate germination when *GID1c* is present, suggesting that *GID1b* may sometimes function as a negative regulator of the downstream positive regulator *GID1c* (Figure 7). The notion that *GID1b* can negatively regulate germination is supported by the fact that the *gid1b* and the *gid1ab* double mutants showed a strong increase in dark seed germination compared to Col WT (Figure 4; Supporting Information Figure S4). The *gid1a* mutation only showed increased dark germination compared to WT under green light or in more dormant seeds. This suggests that *GID1b*, and to a lesser extent *GID1a*, can act as negative regulators of dark seed germination. *GID1c* appears to be a key positive regulator of seed germination. This is interesting given that of the *gid1* single mutants, *gid1c* had the strongest decrease in GA dose–response (Figure 3).

These results were the reverse of what we initially expected based on overexpression experiments (Ariizumi et al., 2013; Hauvermale et al., 2014, 2015). *GID1b* overexpression (*GID1b‐OE*) better rescued the germination of the GA‐insensitive *sly1‐2* mutant than overexpression of *GID1c* and *GID1a*. *GID1b‐OE* also better enhanced the GA sensitivity of the GA biosynthesis mutant *ga1‐3*. It may be that expression on the CaMV 35S promoter does not result in *GID1b* expression at the appropriate time or place for it to function as a negative regulator. We do know that 35S:HA:GID1a, 35S:HA:GID1b, and 35S:HA:GID1c protein expression cannot be detected at all in dormant seeds but is detected in after‐ripened seeds (Hauvermale et al., 2015). An antibody that detected all three GID1 proteins detected low level GID1 protein expression in dormant seeds that increased with after‐ripening. Thus, we suspect that HA:GID1b protein may not be present to block germination of dormant seeds when expressed on the 35S promoter. A second possibility is that *GID1b* cannot function as a negative regulator of germination in the absence of GA and *SLY1*. Future work will need to examine whether GID1 protein function in germination is subject to complex post‐translational regulation. These conflicting results point out the importance of interpreting overexpression results with caution. Another caveat is that the *ga1‐3* and *sly1‐2* mutants are in the Landsberg *erecta* (L*er*) ecotype whereas the *gid1* mutants are in the Col ecotype. Future work will need to examine the effects of *GID1* gene overexpression in the Col ecotype. Ecotype can impact the phenotypes of GA receptor mutants given that the *gid1b‐1* mutation in the Columbia‐0 ecotype showed little or no reduction in GA sensitivity, whereas another *gid1b* allele in the Nossen ecotype showed a strong reduction in GA sensitivity (Figure 3; Iuchi et al., 2007).

Other lines of evidence raised the possibility that the Arabidopsis GA receptors have evolved specialized functions. Based on predicted amino acid sequence alignment, Arabidopsis GID1a more closely resembles GID1c and the sole copy of GID1 in rice (*Oryza sativa*, OsGID1) than GID1b (Nakajima et al., 2006; Yamamoto et al., 2010). GID1a has 85% amino acid identity with GID1c, but only 66% amino acid identity with GID1b (Nelson & Steber, 2016). This difference in amino acid similarity is associated with functional differences in DELLA and GA‐binding activities (Nakajima et al., 2006; Yamamoto et al., 2010). Rice GID1 and GID1a/c‐type receptors absolutely require GA in order to bind DELLA protein, whereas GID1b‐type receptors have a limited ability to bind DELLA without GA that is enhanced by GA hormone. A single amino acid change in the rice OsGID1a (P99A) converted this receptor to a GID1b‐like, GA‐enhanced and GA‐hypersensitive receptor. The GA‐enhanced GA signaling suggests that GID1b is fundamentally different from GID1a and GID1c. The three GA receptors also differ in their affinity for GA hormone. GA associated with GDI1b more quickly than GID1a and GID1c, while GA dissociated from GID1a more slowly than from GID1c and GID1b. It is possible that the stronger rescue of *sly1‐2* germination by *GID1b‐OE* is due to either to a lower GA requirement or to higher DELLA protein affinity. The GID1 transcripts also show differences in mRNA expression during germination (Hauvermale et al., 2015). *GID1a* and *c* transcripts are more strongly induced by cold stratification than *GID1b*, whereas *GID1b* transcript showed higher relative expression levels in the dark. These expression differences initially prompted us to more closely examine the relative function of *GID1* genes during germination.

Future work will need to examine whether *GID1* genes have specialized functions in other plant species or in other GA responses. While monocots tend to have a single *GID1a/c*‐type receptor like rice, sequence analysis showed that multiple eudicot species have both *GID1a/c*‐type and *GID1b*‐type GA receptors such as soybean (*Glycine max*), garden cress (*Lepidium sativum*), tomato (*Solanum lycopersicum*), alfalfa (*Medicago truncatula*), and oilseed rape (*Brassica napus*) (Voegele et al., 2011; Yamamoto et al., 2010). Functional analysis is needed to examine if *GID1b* can act as a negative regulator of seed germination in these plant species. Future work should also explore whether *GID1b* can negatively regulate other GA‐stimulated responses such as cell expansion, transition to flowering, and fertility. *GID1a* and *GID1c* are important positive regulators of stem elongation since the *gid1ac* double mutant is a severe dwarf, whereas the *gid1ab* and *gid1bc* double mutants are not (Griffiths et al., 2006; Willige et al., 2007). Conversely, *GID1a* and *GID1b* play a stronger role in fertility. Interestingly, Griffiths et al., 2006 observed that the *gid1a‐1 gid1b‐1* double mutant had a significantly longer final stem length than Col‐0 WT. Thus, *GID1b* may also play a negative role in stem elongation. Future work should investigate whether *GID1b* may negatively regulate hypocotyl elongation in the dark or effects on red and far red light responses in seeds and seedlings.

Plants can evolve complex hormonal responses by having multiple hormone receptors with specialized functions. For example, it appears that only a subset of the ABA (abscisic acid) hormone receptor genes are involved in stomatal closure, making them good targets for improving drought tolerance (Okamoto et al., 2013; Park et al., 2015). The complex genetic interactions of *GID1* GA receptor mutants are reminiscent of the interactions between the three salicylic acid (SA) receptors, *NPR1*,* NPR3*, and *NPR4* (Fu et al., 2012; Kuai, MacLeod, & Després, 2015; Wu et al., 2012). The *npr1* mutant is pathogen susceptible due to failure to induce PR (Pathogenesis Related) genes, whereas the *npr3 npr4* double mutant is pathogen resistant due to increased systemic acquired resistance (SAR). The *npr1 npr3 npr4* triple has an *npr1*‐like phenotype, indicating that *NPR1* is the downstream regulator. *NPR1* is a transcriptional co‐regulator that activates SAR, but represses hypersensitive response (HR). SAR allows cell survival and pathogen resistance through PR gene expression, whereas HR prevents the spread of biotrophic pathogens through programmed cell death at the initial infection site. HR needs to be limited to the infection site. Both *NPR3* and *NPR4* are adapter proteins for CUL3 E3 ubiquitin ligases that bind to and target NPR1 for destruction. The NPR4 receptor has a high affinity and NPR3 a low affinity for SA hormone. SA‐binding blocks the NPR4‐NPR1 interaction, but stimulates the NPR3‐NPR1 interaction. The current model describes three SA hormone conditions leading to different levels of NPR1 function. (a) In the absence of pathogen, lack of SA causes NPR4 to target NPR1 for destruction leading to lack of SAR. (b) After pathogen attack, low SA levels in cells distant from the infection site block NPR4‐directed NPR1 destruction, leading to NPR1‐stimulated SAR but not HR. (c) At the site of pathogen attack, high SA levels active NPR3‐directed NPR1 destruction, thereby lifting NPR1‐repression of HR causing cell death at the infection site. This elegant model for dose‐dependent signaling allows different responses to pathogen attack depending on SA hormone levels.

By analogy to SA signaling, different GID1 proteins may allow germination to respond to different environmental signals, possibly due to differences in GA hormone levels and/or receptor affinities (Figure 7). For example, the high GA‐affinity receptor GID1b may inhibit germination when GA levels are low (in the dark or in dormant seeds), and the lower GA‐affinity receptor GID1c may stimulate germination when GA levels are high (in the light and after‐ripened or cold‐stratified seeds) (reviewed in Yamaguchi, 2008; Nelson & Steber, 2016). *GID1c* is the key downstream positive regulator of light and dark germination. (Figure 5; Figure 7a,b). Based on the failure of light and dark germination in the *gid1abc* triple mutant, stimulation of dark germination by the *gid1b* and *gid1a* mutations requires the presence of *GID1c* as a positive regulator of germination. The fact that *gid1b* and *gid1c* have additive effects on dark germination of *gid1bc* suggests that *GID1b* negative regulation may be more complex, possibly functioning through an as yet unidentified target. *GID1a* functions independently of *GID1c* as a positive regulator of light and dark germination since *gid1a* and *gid1c* act additively. In light‐germinating dormant seeds, *GID1b* functions as an upstream negative regulator of *GID1c* since *gid1c* is epistatic to *gid1b* (Figure 2; Figure 7a). In the dark, *GID1a* and *GID1b* both function as negative regulators of *GID1c* since the *gid1b* and *gid1ab* double mutants germinate more efficiently than WT. If *GID1a* and *GID1b* did not negatively regulate germination via *GID1c*, then we would expect the *gid1abc* triple to germinate in the dark in a manner similar to *gid1ab* (Figure 7b). Future work will need to examine how *GID1b* negatively regulates *GID1c*. It will be interesting to learn whether the Arabidopsis *GID1* genes have evolved to detect environmental conditions such as the passing of time (after‐ripening), temperature, and lighting conditions in order to ensure seedling survival after seed germination.

## AUTHOR CONTRIBUTIONS

C.M.S provided the initial research design, guidance in performing and analyzing the results of experiments, and funding. W.G. obtained funding, designed and performed experiments, and performed the statistical analyses of results. C.M.S and W.G. wrote and edited the manuscript.
