# Supplementary material for: Positive and negative regulation of seed germination by the Arabidopsis GA hormone receptors, GID1a, b, and c
Source: Plant Direct. 2018 Sep 21;2(9):e00083. doi: 10.1002/pld3.83 (PMC6508844; doi:10.1002/pld3.83)
Supplement: Supplementary file 1 [file PLD3-2-e00083-s001.pdf]

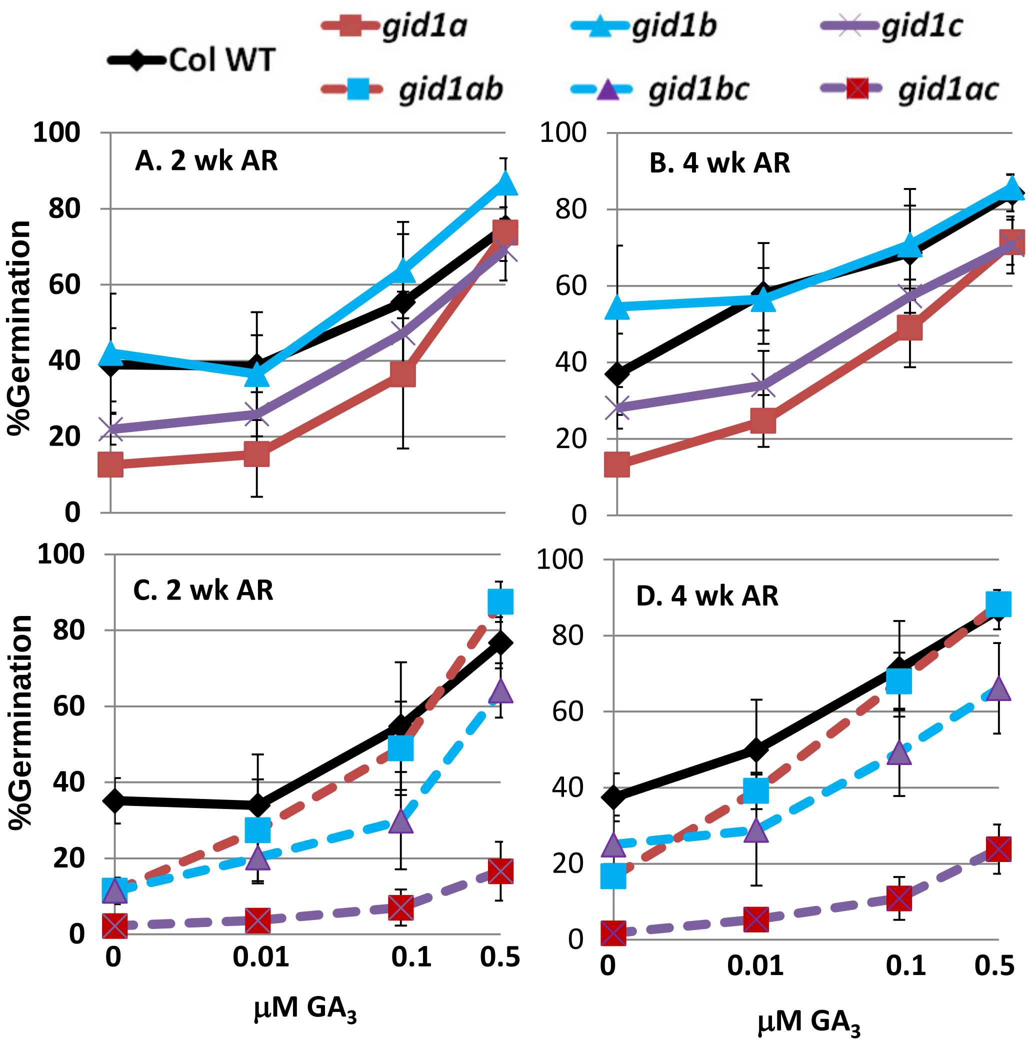

Supplemental Figure S1. GA dose-response of *gid1* mutants. The GA sensitivity of A) and B) *gid1* single mutants (solid lines), and C) and D) double mutants (dashed lines) are shown. Seeds were after-ripened for: A) and C) 2 weeks (wk) and B) and D) 4 wk. Average % germination at day 5 of imbibition on indicated GA concentrations are shown. The x-axis is in log_{10} scale. Error=SD (n=4).

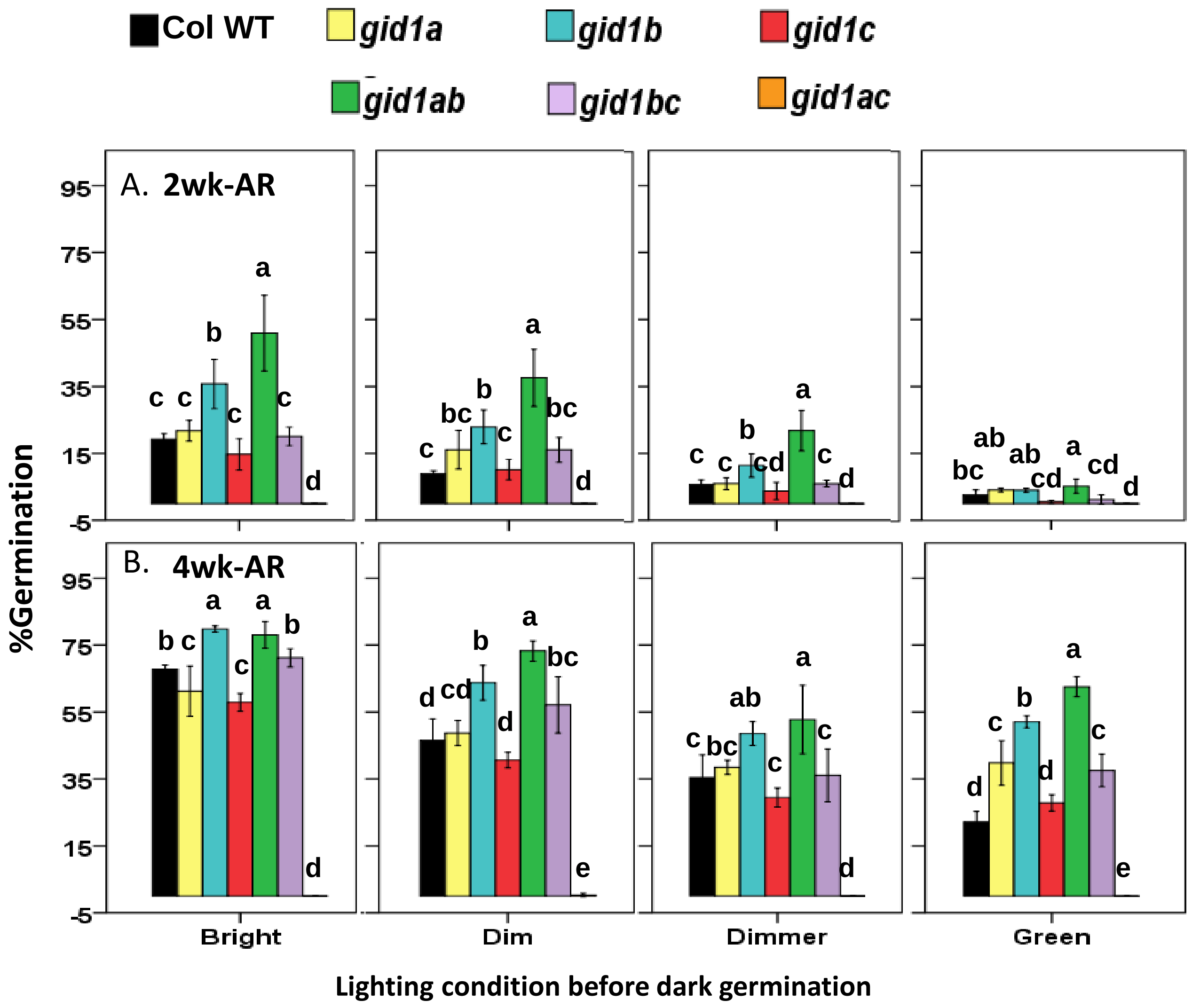

Supplemental Figure S2. Statistically significant differences in *gid1* mutant dark germination. Seeds after-ripened for (A) 2wk and (B) 4wk were plated under bright, dim, or dimmer fluorescent light or green LED light, then incubated in the dark at 22°C for 14 days. Each bar graph shows the genotypes in order: Col WT, *gid1a*, *gid1b*, *gid1c*, *gid1ab*, *gid1bc*, and *gid1ac*. Letters indicate values falling into significantly different statistical classes ($p \leq 0.05$) based on a one-way ANOVA. Error bars = SE (n = 3).

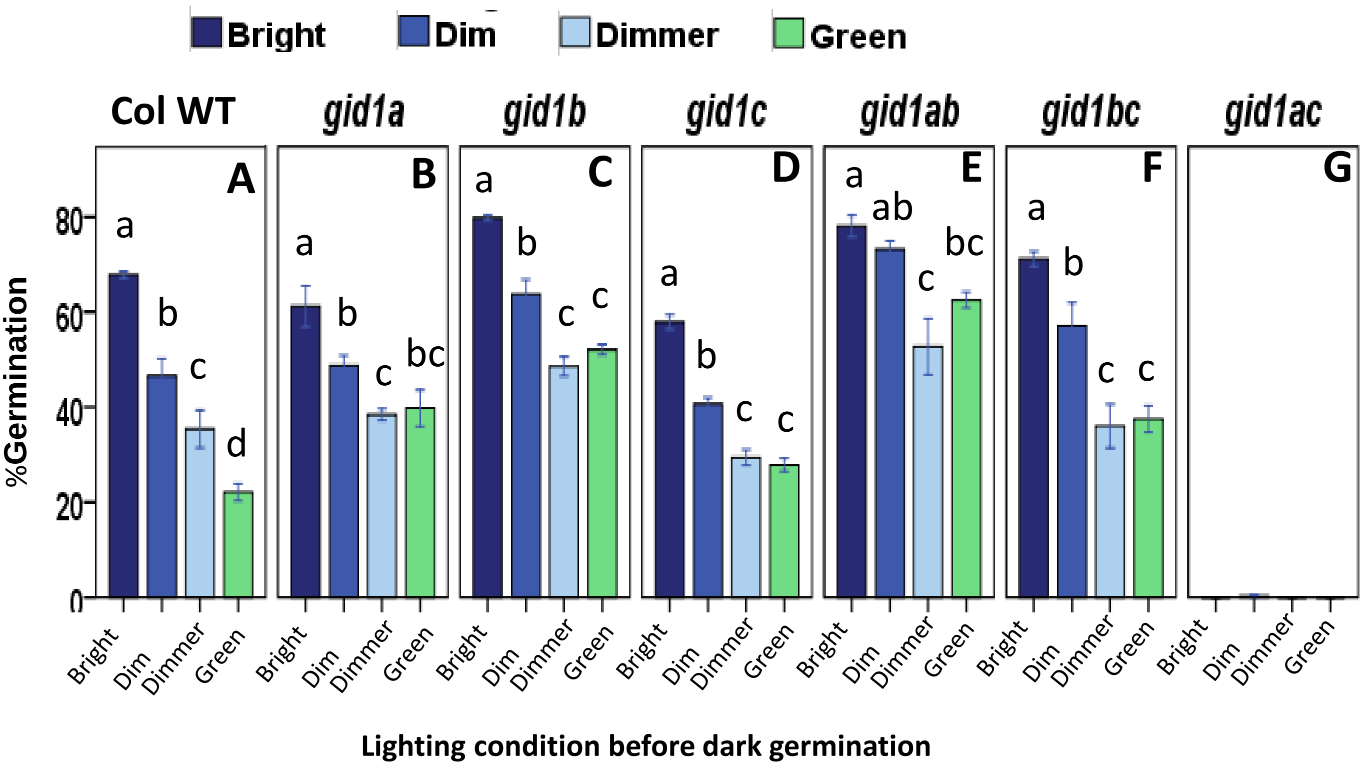

Supplemental Figure S3. Effect of light intensity during plating on *gid1* dark germination. The effects of *gid1* mutants (4wk-AR) on dark germination varied with light intensity during plating. Col WT and each gid1 mutant were showed A-G respectively. Error =SE (n = 3).

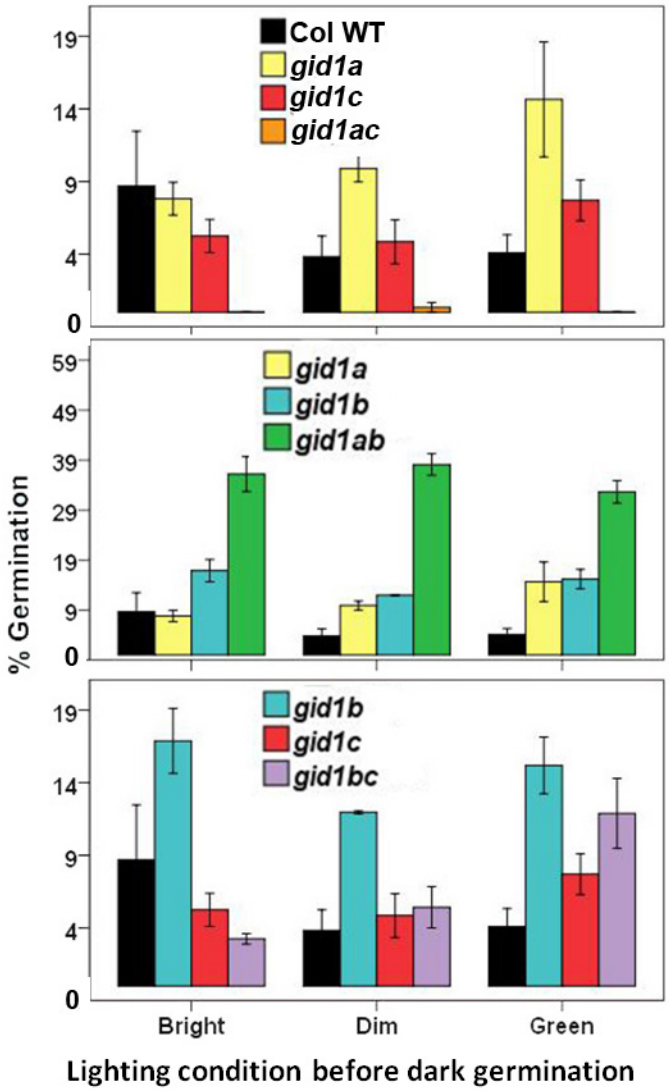

Supplement Figure S4. Dark germination of a second set of *gid1* mutant seeds. The same germination experiment shown in Figure Seeds at 4 wk-AR were sterilized in dim light and plated under "Bright", "Dim" and "Green" lighting conditions. MS-agar plants were incubated for 14 d in the dark at 22°C. Note that y-axes are not to the same scale. Shown is mean $\pm$ SE, n = 3.

Supplemental Table 1. *p*-values from ANOVA of Figure S2, Effect of *gid1* mutants on dark germination, 2wk AR.

**2wk-AR Bright**	Col	*gid1a*	*gid1b*	*gid1c*	*gid1ab*	*gid1bc*	*gid1ac*
Col WT	—	0.593	0.003	0.342	<0.001	0.862	0.001
gid1a		—	0.009	0.148	<0.001	0.717	<0.001
gid1b			—	<0.001	0.005	0.004	<0.001
gid1c				—	<0.001	0.266	0.007
gid1ab					—	<0.001	<0.001
gid1bc						—	0.001
gid1ac							—
**2wk-AR Dim**	Col	*gid1a*	*gid1b*	*gid1c*	*gid1ab*	*gid1bc*	*gid1ac*
Col WT	—	0.081	0.003	0.741	<0.001	0.082	0.038
gid1a		—	0.098	0.146	<0.001	0.994	0.001
gid1b			—	0.005	0.002	0.097	<0.001
gid1c				—	<0.001	0.147	0.020
gid1ab					—	<0.001	<0.001
gid1bc						—	0.001
gid1ac							—
**2wk-AR**	Col	*gid1a*	*gid1b*	*gid1c*	*gid1ab*	*gid1bc*	*gid1ac*
Col WT	—	0.918	0.033	0.431	<0.001	0.929	0.032
gid1a		—	0.041	0.375	<0.001	0.988	0.026
gid1b			—	0.007	0.001	0.040	<0.001
gid1c				—	<0.001	0.383	0.140
gid1ab					—	<0.001	<0.001
gid1bc						—	0.027
gid1ac							—
**2wk-AR Green**	Col	*gid1a*	*gid1b*	*gid1c*	*gid1ab*	*gid1bc*	*gid1ac*
Col WT	—	0.143	0.153	0.053	0.016	0.185	0.018
gid1a		—	0.976	0.003	0.250	0.011	0.001
gid1b			—	0.003	0.234	0.012	0.001
gid1c				—	<0.001	0.487	0.584
gid1ab					—	0.001	<0.001
gid1bc						—	0.223
gid1ac							—

Red font indicates significant *p*-values.

Supplemental Table 2. *p*-values from ANOVA of Figure S2, Effect of *gid1* mutants on dark germination, 4 wk AR.

**4wk-AR Bright**	Col	*gid1a*	*gid1b*	*gid1c*	*gid1ab*	*gid1bc*	*gid1ac*
Col WT	—	0.039	0.001	0.004	0.003	0.271	<0.001
gid1a		—	<0.001	0.275	<0.001	0.004	<0.001
gid1b			—	<0.001	0.557	0.010	<0.001
gid1c				—	<0.001	<0.001	<0.001
gid1ab					—	0.033	<0.001
gid1bc						—	<0.001
gid1ac							—
**4wk-AR Dim**	Col	gid1a	gid1b	gid1c	gid1ab	gid1bc	gid1ac
Col WT	—	0.591	0.001	0.160	<0.001	0.019	<0.001
gid1a		—	0.002	0.061	<0.001	0.054	<0.001
gid1b			—	<0.001	0.032	0.119	<0.001
gid1c				—	<0.001	0.001	<0.001
gid1ab					—	0.001	<0.001
gid1bc						—	<0.001
gid1ac							—
**4wk-AR**	Col	gid1a	gid1b	gid1c	gid1ab	gid1bc	gid1ac
Col WT	—	0.537	0.015	0.229	0.003	0.897	<0.001
gid1a		—	0.052	0.080	0.010	0.623	<0.001
gid1b			—	0.001	0.397	0.020	<0.001
gid1c				—	<0.001	0.187	<0.001
gid1ab					—	0.004	<0.001
gid1bc						—	<0.001
gid1ac							—
**4wk-AR Green**	Col	gid1a	gid1b	gid1c	gid1ab	gid1bc	gid1ac
Col WT	—	<0.001	<0.001	0.079	<0.001	<0.001	<0.001
gid1a		—	0.001	0.001	<0.001	0.470	<0.001
gid1b			—	<0.001	0.004	<0.001	<0.001
gid1c				—	<0.001	0.006	<0.001
gid1ab					—	<0.001	<0.001
gid1bc						—	<0.001
gid1ac							—

Red font indicates significant *p*-values

Supplement Table 3. *p*-values from ANOVA of Figure S3, Effect of light intensity on *gid1* dark germination.

**4wk-AR Col**	Bright	Dim	Dimmer	Green
Bright	—	0.001	<0.001	<0.001
Dim		—	0.025	<0.001
Dimmer			—	0.011
Green				—
**4wk-AR gid1a**	Bright	Dim	Dimmer	Green
Bright	—	0.023	0.001	0.001
Dim		—	0.049	0.079
Dimmer			—	0.769
Green				—
**4wk-AR gid1b**	Bright	Dim	Dimmer	Green
Bright	—	<0.001	<0.001	<0.001
Dim		—	<0.001	0.002
Dimmer			—	0.226
Green				—
**4wk-AR gid1c**	Bright	Dim	Dimmer	Green
Bright	—	<0.001	<0.001	<0.001
Dim		—	0.001	<0.001
Dimmer			—	0.483
Green				—
**4wk-AR**	Bright	Dim	Dimmer	Green
Bright	—	0.347	0.001	0.012
Dim		—	0.003	0.057
Dimmer			—	0.076
Green				—
**4wk-AR**	Bright	Dim	Dimmer	Green
Bright	—	0.027	<0.001	<0.001
Dim		—	0.004	0.006
Dimmer			—	0.781
Green				—
**4wk-AR**	Bright	Dim	Dimmer	Green
Bright	—	0.195	1.000	1.000
Dim		—	0.195	0.195
Dimmer			—	1.000
Green				—

Red font indicates significant *p*-values
