# Supplementary material for: Positive and negative regulation of seed germination by the Arabidopsis GA hormone receptors, GID1a, b, and c
Source: Plant Direct. 2018 Sep 21;2(9):e00083. doi: 10.1002/pld3.83 (PMC6508844; doi:10.1002/pld3.83)
Supplement: Supplementary file 2 [file PLD3-2-e00083-s002.pdf]

Response to review of Plant Direct: 2018-00169

Authors: Wenjing Ge and Camille M. Steber
Title: Positive and negative regulation of seed germination by the Arabidopsis GA hormone receptors, GID1a, b, and c

The authors wish to thank the reviewers and the editor for their insightful comments on the manuscript. Our responses to each comment are given below.

Reviewer 1

Comment 1. Because the gid1bc double mutant germinates better than gid1c (Figure S2 and lines 330-331) there must be additional negative regulation that is not shown in Figure 7. This is probably worth mention somewhere in the manuscript
Response 1. The gid1bc double mutant germinates more efficiently than gid1c but less well than gid1b. This additive effect suggested that gid1b and gid1c act in parallel to negatively and positively regulate germination. However, Figure 7 does not show them acting entirely in parallel because a second result contradicted this notion. If gid1b and gid1c act in parallel, then the gid1abc triple should have germinated in a manner similar to gid1ab in the dark. It didn't, suggesting that a mutation in the negative regulator GID1b is not sufficient to stimulate germination unless there is positive regulation by GID1c. This is why Figure 7 shows GID1b negatively regulating germination via GID1c rather than in parallel to GID1c. Instead, Figure 7 shows GID1b acting weakly as a positive regulator of germination in parallel with GID1c. This is because the gid1abc triple completely fails to germinate, suggesting that all three GID1 genes can stimulate dark germination. It also suggests that negative regulation by GID1b and GID1a is via GIDc, the downstream positive regulatory (lines 349-352).

Since gid1b and gid1c had an additive effect, the reviewer rightly pointed out that negative regulation by GID1b is likely more complex than shown in the Figure 7 model. To point out the possibility that there may be something more going on with GID1b negative regulation, we added a sentence to the Discussion section lines 521-523. "The fact that gid1b and gid1c have additive effects on dark germination of gid1bc suggests that GID1b negative regulation may be more complex, possibly functioning through an as yet unidentified target."

Comment 2. Please list the enzyme and source used for PCR (lines 162-164).
Response 2. The fact that NEB Taq was used was added to the Methods line 162. Based on this comment we checked for other places where we may have left out the manufacturer. The commercial source of GA3 hormone was added to the Methods on line 193.

Comment 3. This becomes clear as one reads further but it would be helpful to indicate in the heading (line 233), or first sentence of the section that the experiments are examining germination in light.
Response 3. The term "light germination" was added to the headings on both line 233 and on line 270.

Comment 4. The statement in lines 306-307 should be modified to more accurately describe the gid1c phenotype. Also because it has the statistical analysis, Figure S2 rather than S3 should be cited. Figure S2 shows that the gid1c mutant has reduced germination (bright) or no significant change (dim, dimmer and green) relative to WT.
Response 4. The fact that gid1c showed a significant difference in dark germination from WT only after plating under bright lighting was added to the sentence on lines306-307, and Figure S2 was cited instead of Figure S3.

Comment 5. The sentences in lines 349-351 and lines 517 and 518 are confusing.
Response 5. The awkward wording in lines 349-352 and lines 519-521 were corrected. This should, hopefully, also help clarify the model in Figure 7 as explained in the response to comment 1.

Comment 6. Figure 6 legend. It is helpful to mention that this is showing germination in light here.
Response 6. Figure 6 is not germination data. This is seed size in mg/500 seeds as indicated in the y-axis label.

Comment 7. There is no mention of the supplementary tables in the text.
Response 7. Supplemental tables 1 and 2 are now referenced on line 299.

Reviewer 2

Comment 1. Line 108/109: Correct citations.
Response 1. Corrected citations so that they no longer include the complete references.

Comment 2a. Labeling of x- and y-axes of all figures is rather odd. Shouldn't it be "Germination [%]" instead of "% Germination" and "Time [days]" instead of "Days".
Response 2a. The x and y axis labels were left unaltered with the editor's permission.

Comment 2b. I also propose to use a log x-axis scale in Figure 3 and S1.
Response 2b. This is a good suggestion. The figures were updated.

Comment 2c. Figures 4, S2-S4 have no X-axis labeling at all, need to say what it is there.
Response 2c. The x-axis labels for these figures were category names. The reviewer is right to point out that these categories can be confusing if the reader has not yet seen the explanation of the experiment in the figure legend. The x-axis label "Lighting condition before dark germination" was added, and further explanation was added to the Figure 4 legend.

Comment 3. A recent review on GA in seeds should be cited: Urbanova T, Leubner-Metzger G Gibberellins and seed germination. Chapter 9, pp. 253-284 (2016)
In: Hedden P, Thomas SG (Eds). The Gibberellins. Annual Plant Reviews Volume 49. Wiley-Blackwell Publishing Ltd., Oxford, United Kingdom.
Response 3. This is a good point. A citation of the recommended article was added to line 91.
